# Human Cytomegalovirus Cell Tropism and Host Cell Receptors

**DOI:** 10.3390/vaccines7030070

**Published:** 2019-07-22

**Authors:** Giuseppe Gerna, Anna Kabanova, Daniele Lilleri

**Affiliations:** 1 Laboratories of Genetics, Transplantology and Cardiovascular Diseases, Fondazione IRCCS Policlinico San Matteo, 27100 Pavia, Italy; 2 Toscana Life Sciences Foundation, 53100 Siena, Italy

**Keywords:** HCMV, epithelial cells/endothelial cells, cell tropism, cellular receptors, PDGFRα, Nrp2

## Abstract

In the 1970s–1980s, a striking increase in the number of disseminated human cytomegalovirus (HCMV) infections occurred in immunosuppressed patient populations. Autopsy findings documented the in vivo disseminated infection (besides fibroblasts) of epithelial cells, endothelial cells, and polymorphonuclear leukocytes. As a result, multiple diagnostic assays, such as quantification of HCMV antigenemia (pp65), viremia (infectious virus), and DNAemia (HCMV DNA) in patient blood, were developed. In vitro experiments showed that only low passage or endothelial cell-passaged clinical isolates, and not laboratory-adapted strains, could reproduce both HCMV leuko- and endothelial cell-tropism, which were found through genetic analysis to require the three viral genes UL128, UL130, and UL131 of the HCMV UL128 locus (UL128L). Products of this locus, together with gH/gL, were shown to form the gH/gL/pUL128L pentamer complex (PC) required for infection of epithelial cells/endothelial cells, whereas gH/gL and gO form the gH/gL/gO trimer complex (TC) required for infection of all cell types. In 2016, following previous work, a receptor for the TC that mediates entry into fibroblasts was identified as PDGFRα, while in 2018, a receptor for the PC that mediates entry into endothelial/epithelial cells was identified as neuropilin2 (Nrp2). Furthermore, the olfactory receptor family member OR14I1 was recently identified as a possible additional receptor for the PC in epithelial cells. Thus, current data support two models of viral entry: (i) in fibroblasts, following interaction of PDGFRα with TC, the latter activates gB to fuse the virus envelope with the cell membrane, whereas (ii) in epithelial cells/endothelial cells, interaction of Nrp2 (and OR14I1) with PC promotes endocytosis of virus particles, followed by gB activation by gH/gL/gO (or gH/gL) and final low-pH entry into the cell.

## 1. Introduction

Following the advent and the rapid expansion of human cytomegalovirus (HCMV) infections caused by both human immunodeficiency virus (HIV) epidemics, as well as the administration of immunosuppressive drugs required for organ and stem cell transplantation, the rate of disseminated HCMV infections reached a peak, thus giving rise to the study of their pathogenesis. Using a double-staining method, including one monoclonal antibody (mAb) staining the cell type and another staining the HCMV gene products, the cells most frequently identified as permissive to HCMV in vivo in different organs of patients with disseminated infection were found to be endothelial cells, epithelial cells, human fibroblasts, and smooth muscle cells. Of the hematogenous cells, monocytes, macrophages, and dendritic cells from the myeloid lineage were found to be permissive to HCMV, whereas no cells from the lymphoid lineage were [1,2,3]. Polymorphonuclear leukocytes (PMNLs) do not seem to be permissive to HCMV replication, but can transport the virus passively [4]. One important cellular reservoir of latent HCMV is CD34^+^ hematopoietic progenitor cells resident in the bone marrow [5].

## 2. HCMV-Infected Endothelial Cells and Leukocytes In Vivo

**Figure 1:**
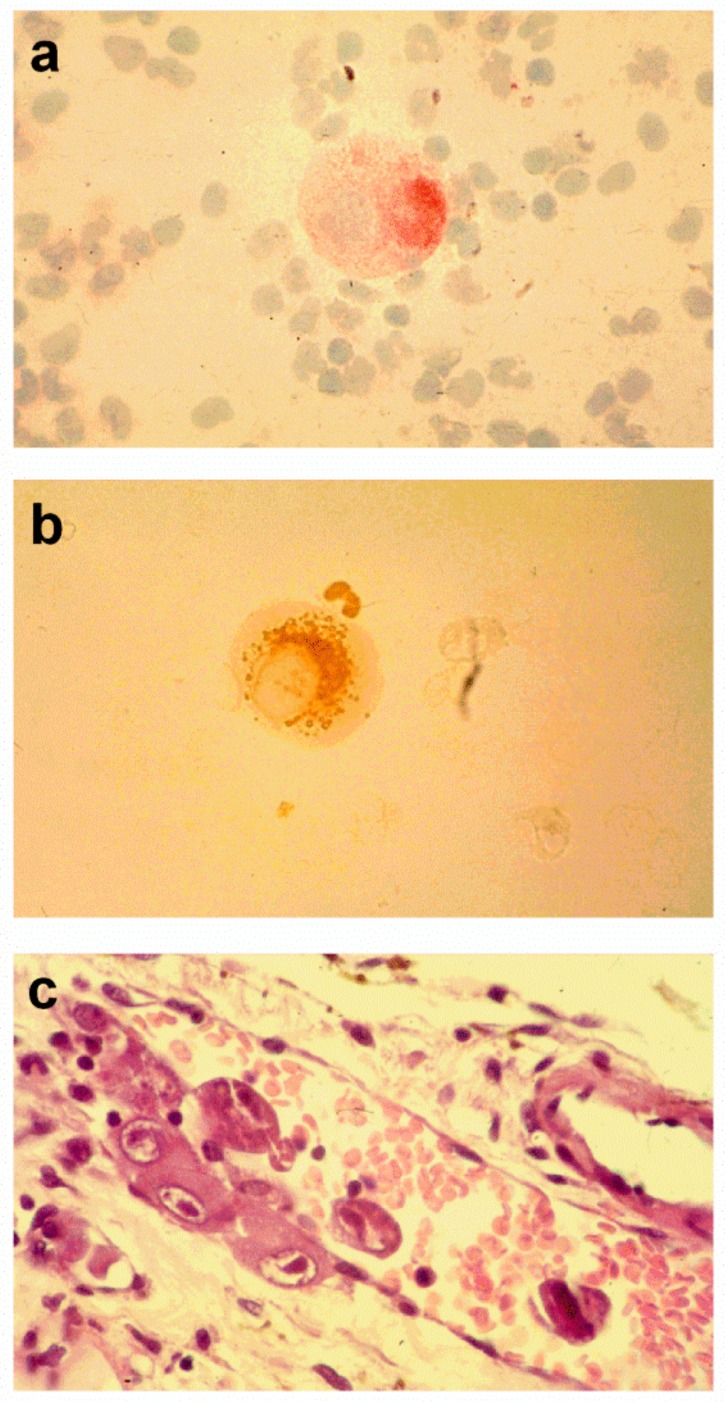
Circulating cytomegalic endothelial cells stained with: (**a**) the endothelial cell-specific PAL-E monoclonal antibody; (**b**) a pp65-specific human cytomegalovirus (HCMV) monoclonal antibody (a pp65-positive polymorphonuclear leukocyte is shown in close proximity). (**c**) Several cytomegalic endothelial cells are present along the vessel wall and in the bloodstream of a prostatic vessel of an AIDS patient with disseminated HCMV infection (from [12]).

In immunosuppressed patients, HCMV can cause either disseminated infection or end-organ disease. The extent of HCMV infection of endothelial cells in vivo was shown in findings on histological sections from nearly all organs of patients who died of AIDS and disseminated HCMV infection [4,6]. HCMV-infected endothelial cells were widespread throughout the body along the vessel walls; they often invaded the vessel lumen and detached, thus entering the blood stream as circulating cytomegalic endothelial cells (CCECs), which were often detected in the peripheral blood of immunocompromised patients with disseminated infection (Figure 1) [7]. In addition, evaluation by electron microscopy (EM) revealed that CCECs in these patients were generally associated with end-organ disease and were found to contain mature virus particles [7,8,9]. 

Concomitant with the presence of CCECs, generally in even greater proportions, two subpopulations of peripheral blood leukocytes (PBL), i.e., polymorphonuclear leukocytes (PMNLs) and monocytes, were found to carry infectious HCMV and virus products in the blood of patients with disseminated infection [10]. The ability of both PMNLs and monocytes to carry infectious viruses and spread viral infections was shown by the consistent virus recovery from leukocytes drawn ex vivo from immunocompromised patients, following co-culture with susceptible endothelial cells or fibroblasts. This finding documented that virus uptake by leukocytes did not result in virus degradation. Conversely, it was shown that HCMV replication in both PMNLs and monocytes was blocked after immediate-early (IE) antigen synthesis [11]. Thus, it was concluded that infectious virions were passively transported by leukocytes [11]. Based on the results reported above, there is strong evidence that both CCECs and PMNLs contribute directly to disseminated infection. 

Taken together, the data reported above allowed us to propose a model for the study of the interaction between infected endothelial cells and leukocytes [13]. According to this model, following chemoattraction of PMNLs by infected endothelial cells through a virus-encoded α-chemokine (or host-encoded or a combination of both) and attachment to endothelial cells through the interaction between CD18 and the intercellular adhesion molecule-1 (ICAM-1), discrete points of fusion between adjacent cellular membranes are established, allowing virus and viral products to be transferred to PMNLs [11]. Then, PMNLs carrying the infectious virus can detach from endothelial cells and be transported through the bloodstream to newly-uninfected endothelial cells of blood vessels, thus transmitting the infection to distant sites. 

These observations prompted the need for more rapid diagnostic assays than virus culture to monitor HCMV disseminated infection in immunocompromised patients. The first assay, developed at the end of the 1980s, was HCMV antigenemia, i.e., the assay that employs cytospin preparations to detect peripheral blood leukocytes (PBLs) that are positive for a viral protein. This protein was initially thought to be the immediate-early protein p72 [14,15], but was then identified as the lower matrix phosphoprotein pp65 present in leukocytes as a result of the uptake of viral dense bodies, as well as infectious virions and non-infectious enveloped virus particles [15,16]. The second assay was HCMV viremia, i.e., the assay capable of recovering infectious viruses from PBLs, following their co-culture with fibroblasts [17,18]. This method provided a rapid diagnostic tool for the quantification of infectious virus in blood in less than 24 h and a rapid evaluation of the efficacy of antiviral treatment in patients with disseminated infection, except in cases where a drug-resistant strain was emerging.

A third assay, developed at the beginning of the 1990s by several groups, was the polymerase chain reaction (PCR) for the quantification (QPCR) of HCMV DNA in blood leukocytes (leukoDNAemia) and plasma (plasmaDNAemia) or whole blood (DNAemia). For several years, it was possible to monitor HCMV infections closely in immunocompromised patients by simultaneously performing antigenemia, viremia, and DNAemia assays [19]. The simultaneous quantification of viral DNA in PBL and plasma documented that the DNAemia level was higher in PBL than in plasma [20]. In addition, DNAemia was shown to correlate better with clinical symptoms than antigenemia and viremia did [21]. Finally, two additional assays were developed, both of which were related to the HCMV properties of endothelial cell- and leuko-tropism: the nucleic-acid sequence-based amplification (NASBA) technique for the detection of both IE and late mRNAs [22,23]; and the quantification of CCECs (mentioned above) in cytospin preparations to determine antigenemia [7]. 

Overall, these findings suggest a critical role of endothelial cell- and leuko-tropism in the HCMV pathogenic potential in vivo and its dissemination throughout the body. 

## 3. Interaction of Leukocytes and HCMV-Infected Endothelial Cells In Vitro 

The in vivo findings reported above, as well as previous reports claiming bidirectional transmission of infectious cytomegalovirus between monocytes and vascular endothelial cells [24], prompted us to investigate the transmission of HCMV from infected endothelial cells to leukocytes (both PMNLs and monocytes) in vitro. However, our efforts to reproduce in vitro antigenemia failed repeatedly for five years, during which time we were using laboratory-adapted HCMV strains such as AD169, Towne, or even Toledo. The first successful attempt occurred in 1998, when, based on previous observations that endothelial cell-propagated strains maintain endothelial cell pathogenicity [25], we used recent clinical isolates instead of laboratory strains to infect endothelial cells [26]. From the technical standpoint, PBL from healthy blood donors were co-cultured with HCMV-infected endothelial cells (or fibroblasts) and then separated by means of a transwell migration assay, in which purified leukocytes (PMNLs and monocytes), attracted by the potent chemoattractant N-formylmethionine leucyl-phenylalanine (FMLP), migrated to the lower chamber of the transwell device. In parallel with antigenemia, also viremia, DNAemia, and RNAemia were experimentally reproduced in vitro. In other words, leukocytes positive for pp65 were also positive for viral DNA and RNA, as well as the infectious virus [11]. 

The pathogenetic mechanism underlying the transfer of virus and viral products from infected endothelial cells to leukocytes seems to occur through the following sequential steps: attraction, adhesion, membrane fusion, and then transfer. Attraction to infected endothelial cells may be mediated by the granulocyte-attractant action of cellular chemokines, such as IL-8 and Groα, which are produced by infected endothelial cells [27]. The next step is adhesion between leukocytes and infected endothelial cells, possibly mediated by the interaction between ICAM-1 on infected endothelial cells and its ligands, the leukocyte function-associated antigen (LFA)-1 on PMNLs, and Mac-1 on monocytes. Both ligands belong to the CD11/18 integrin subfamily. When cell-to-cell contact/adhesion was immunologically prevented, no transfer occurred of virus or virus products from endothelial cells to either PMNLs or monocytes [11,24]. Finally, virus transmission to leukocytes requires microfusion of the two adhering cells. In 2004, a locus in the HCMV genome was identified (see below), which appeared to be essential for both infection of endothelial cells (endothelial cell-tropism) and leukocytes (leuko-tropism).

## 4. HCMV Infection of Epithelial Cells 

Apart from endothelial cells and leukocytes, epithelial cells are the cell type most commonly infected in vivo in patients with disseminated HCMV infection. Autopsy findings on AIDS patients who died of disseminated infection showed that epithelial cells are the ones that are predominantly infected in lungs, gastrointestinal tract, secretory glands, and kidneys [1,2]. In addition, cytomegalic epithelial cells detected in several body fluids, such as broncho-alveolar lavage fluid, urine, saliva, and stools, have been found to derive from their detachment from the basal membrane of the relevant tissues [28]. This wide virus dissemination in vivo was somewhat paralleled in vitro by the susceptibility to HCMV infection of several types of epithelial cells from different tissues, such as retinal pigmented epithelial cells [29], kidney epithelial cells [30], and hepatocytes [31]. Moreover, the susceptibility of epithelial cells to complete HCMV replication occurred only with wild-type recently-isolated HCMV strains, whereas infection with laboratory strains was blocked at the IE phase. 

## 5. Genetic Determinants of Endothelial Cell/Myeloid Cell Tropism

At the beginning of the 2000s, the conviction grew that some genetic mutation must have occurred in the HCMV genome during propagation of laboratory-adapted strains in fibroblast cultures. This assumption was strengthened by the observation that any new clinical isolate extensively propagated in vitro in fibroblasts led to the selection of virus variants that were neither able to transfer virus and viral products from infected cells to leukocytes, nor to grow in endothelial cells [32], resulting in the loss of both endothelial cell- and leuko-tropism. Based on the observation that this was a hallmark of all laboratory-adapted HCMV strains, as well as fibroblast-passaged clinical isolates, we proposed endothelial cell- and leuko-tropism as surrogate markers of pathogenicity for HCMV virulent strains, while their absence should be considered a marker of virus attenuation [33].

In the course of this line of investigation, it was surprising to find that both properties were recovered in the Towne and AD169 laboratory-propagated viral strains, following adaptation to growth in endothelial cells. Interestingly, endothelial cell-tropism was restored much earlier than leuko-tropism [34,35]. The identity of the laboratory and endothelial cell-tropic variants of both AD169 and Towne strains suggested that even a minor variation of the viral genome (e.g., mutation reversion) was likely to be responsible for the loss/rescue of the tropism. Furthermore, the Toledo strain, at that time considered to be a prototype of wild-type HCMV strains, was shown to lack both endothelial cell- and leuko-tropism (just like laboratory-adapted HCMV strains), whereas the endothelial cell-tropic Toledo variant possessed a highly-divergent genome, suggesting that it arose from an unrelated strain [36].

The investigation of the genetic determinants of endothelial cell- and leuko-tropism started in 2002 with a joint project by Italian and German researchers. By cloning an endothelial cell-tropic HCMV isolate (VR1814) as a bacterial artificial chromosome, they generated a stable genetic material that was used in mutagenesis screens [37,38]. Initially, the researchers’ attention was steered towards the unique long b’ (ULb’) region of the HCMV genome as the prime candidate for identifying genetic determinants of endothelial cell- and leuko-tropism. First, it was found that, unlike for the murine CMV, HCMV UL45 (ribonucleotide reductase homolog) was not the genetic determinant of HCMV endothelial cell-tropism, since it was dispensable for the in vitro growth of HCMV in endothelial cells [38]. Secondly, the loss of endothelial cell- and leuko-tropism in AD169 and Towne strains was associated with (but was not due to) the loss of most of the ULb’ region (which moreover is true only for the Towne varS variant, but not in the varL variant [39]). Subsequently it was discovered that the reason behind this was a small frameshift mutation in UL130 (Towne) or UL131 (AD169), which are placed close to the ULb’ locus [40,41]. Thirdly, the low-passage clinical Toledo strain, which was unable to infect endothelial cells, displayed an ULb’ inversion with respect to recent clinical isolates. Finally, an endothelial cell-tropism-deficient plaque-purified variant carrying a UL132-130 deletion within the ULb’ region was selected from a recent endothelial cell-tropic clinical isolate following its propagation in fibroblasts [32]. 

During the course of a two-year study, it became evident that it was the UL128-131 locus (UL128L) that was essential for both HCMV endothelial cell- and leuko-tropism [42]. Several loss-of-function and gain-of-function experiments corroborated this conclusion. Firstly, the introduction of targeted mutations into the UL128L consistently caused loss of HCMV tropism, whereas any mutation introduced into any other gene of ULb’ did not affect it. The second loss-of-function proof consisted of the detection of spontaneous mutations of natural viral variants within UL128L, which were detrimental to both endothelial cell- and leuko-tropism. In particular, eight independent HCMV clinical strains that had extensively propagated in fibroblast exhibited a series of spontaneous mutations, each affecting the coding sequence of one gene within the UL128L. 

On the other hand, gain-of-function was documented by two experimental findings. The first consisted of the phenotypic reversion of multiple HCMV natural variant strains to both endothelial cell-tropism and leuko-tropism following reversal of mutations within the UL128L. This was routinely achieved through the propagation of fibroblast-passaged mutated strains in endothelial cells, with the rescue of the original coding sequence. Conversely, none of the three HCMV mutants with a complete knock-out (KO) of individual UL128L genes was able to re-acquire the ability to grow in endothelial cells, thus proving the essential role of the entire UL128L. The second finding was the partial rescue of both properties by trans-complementation with each of the three individual genes of UL128L. Partial rescue refers to the finding that, of the three single-gene KO mutants of the UL128L, only ∆UL128KO showed selective inactivation of UL128, and was rescued by the autologous gene in trans-complementation experiments, whereas ∆UL130 and ∆UL131 appeared to behave functionally as UL128-131 KO mutants. The reason for this apparently unexplained result came from the Northern blot analysis, showing that the three genes of the UL128L are all part of a single transcriptional unit [42]. Thus, deletion of UL131 also impairs the transcription of the downstream genes UL130 and UL128, and deletion of UL130 impairs transcription of UL128. Conversely, deletion of UL128 preserves the transcription of the upstream genes UL131 and UL130 and can be rescued by trans-complementation with UL128.

## 6. Genetic Determinants of Epithelial Cell-Tropism 

After 2004, when the indispensable role of UL128L genes for endothelial cell-tropism was reported, several research groups investigated their role in epithelial cell-tropism. First of all, it was shown that the UL131 ORF, and presumably the entire UL128L, was required for efficient infection of Retinal Pigmented Epithelial Cells (ARPE)-19 retinal pigmented epithelial cells by the rescued UL131 gene of the laboratory strain AD169, as well as by recent clinical HCMV isolates [43]. Thereafter, the same authors documented that, while the envelope of the laboratory strain AD169 virions contains only the gH/gL/gO glycoprotein complex, the AD169 virus with a repaired UL131 gene carries two distinct gH/gL-containing complexes: gH/gL/gO, mediating infection of fibroblasts, and gH/gL/pUL128-131, which is likely to mediate the infection of both epithelial cells and endothelial cells [44]. The presence of two functionally-distinct gH/gL-containing complexes recalled a similar property of two other human herpesviruses, i.e., Epstein-Barr virus (EBV) [45] and human herpesvirus-6 (HHV-6) [46], both of which use two gH/gL-containing complexes interchangeably to infect different cell types. Furthermore, it was shown that HCMV entry into epithelial cells and endothelial cells depends on genes belonging to the UL128-UL150 cluster and occurs by endocytosis and low pH-dependent fusion, unlike the pH-independent fusion of the virus envelope with the plasma membrane that occurs in fibroblasts [47].

The mechanism of virus entry was definitively clarified in 2008 when, using HCMV mutants lacking UL128-131 gene products and non-replicating adenovirus vectors expressing gH, gL, pUL128, pUL130, and pUL131, it was shown that the export of gH/gL-containing complexes from the endoplasmic reticulum (ER) to the Golgi apparatus and cell surface was significantly increased when all three UL128, UL130, and UL131 gene products were co-expressed with gH/gL. Thus, it was concluded that all three gene products must bind to gH/gL in order to produce the gH/gL/pUL128-131 pentameric complex (PC) utilized for entry into epithelial cells and endothelial cells [48]. In addition, the three ORFs of UL128L were found to be highly conserved in vivo with over 90% nucleotide conservation of all three ORFs [49]. These results were further extended when it was demonstrated that the expression of the PC in ARPE-19 epithelial cells, but not in fibroblasts, caused resistance to HCMV infection [50]. Taken together, these findings confirmed the important role of the PC in endothelial cell-tropism of HCMV and, importantly, anticipated the presence of a pentamer receptor in epithelial cells. 

## 7. Genetic Determinants of HCMV-Tropism for Human Fibroblasts

Since its first isolation in the 1950s, HCMV has been routinely recovered from and propagated in fibroblast cells. However, while the gH/gL/pUL128L PC is required for infection of both epithelial cells and endothelial cells, it is dispensable for infection of fibroblasts. Infection of fibroblasts, in contrast, and probably infection of all cell types by cell-free HCMV, requires gH/gL to bind to another glycoprotein, gO, to form the gH/gL/gO trimer complex (TC), as reported [51,52]. While co-expression of gH, gL, and gO in insect cells using a recombinant baculovirus did not produce detectable gH/gL/gO TC, gH/gL/gO complexes were produced in a mammalian cell system using triple plasmid transfection [52]. In addition, it was found that entry of HCMV into fibroblasts critically depends on the β1 and β3 integrins [53] and occurs by pH-independent direct fusion of the viral envelope with the plasma membrane, unlike pH- and endocytosis-dependent HCMV entry into epithelial cells/endothelial cells [47]. However, the mechanism of entry into fibroblasts is still under discussion. Recent observations suggest that it may occur via a rapid micropinocytosis [54] or even by direct fusion with the outer plasma membrane through the fusogenic activity of gB [55].

## 8. gH/gL/pUL128L and gH/gL/gO Complexes: Development and Characterization of Human Monoclonal Antibody Response

The discovery that HCMV uses different glycoprotein complexes for entry into endothelial cells/epithelial cells [42,43] and into fibroblasts [52] prompted us to investigate whether human antibody response to natural HCMV infection displayed different levels of neutralization potential against the infection of these two cell types. Results showed that in the convalescent phase of primary HCMV infection, the reciprocal geometric mean neutralizing antibody titer was >30-times more potent in neutralizing infection of epithelial cells/endothelial cells than that of fibroblasts [55]. The primary candidates for this differential neutralizing activity appeared to be antibodies directed against UL128L proteins, as indirectly suggested by murine and rabbit polyclonal antibodies raised against the individual components of pUL128L [44,56,57]. In addition, results of vaccination with either the Towne vaccine or recombinant gB adjuvanted with MF59 (gB/MF59) showed that both vaccines elicited epithelial cell-specific neutralizing antibodies (nAbs), which were on average about 30-fold (Towne) or 15-fold (gB) lower than those induced by natural infection [58]. These results were due to the presence of a mutation in the UL128L of Towne, while gB was found to possess a much lower potency in eliciting nAbs compared to PC [59,60,61].

Direct confirmation of these findings was obtained in 2010, when the combination of memory B-cell immortalization and nAb determination in different target cells allowed researchers to identify two panels of human nAbs induced by natural infection [59]. The first panel included mAbs directed to the previously-known targets of the neutralizing activity: gB, gH, and gM/gN. These mAbs displayed similar neutralizing activity (within the nanomolar range of concentration) on both epithelial cells/endothelial cells and fibroblasts. The second panel included numerous mAbs that displayed neutralizing activity at picomolar concentrations, specifically on epithelial cells/endothelial cells, but which lacked neutralizing activity on fibroblasts. mAbs of this second group possessed two properties: (i) they reacted mostly with antigenic sites expressed by two or more genes within UL128L, and in particular the UL130+UL131 dimer; (ii) owing to the high degree of amino acid conservation among UL128L gene products of different virus strains (>90%, [49]), they were able to neutralize different HCMV isolates at a comparable titer.

Structural and biochemical characterization of HCMV TC and PC by mass spectrometry and mutagenesis analysis showed that TC and PC form two mutually-exclusive cell entry complexes [62]. It was found that complex assembly is promoted by the formation of disulfide bonds between the cysteine 144 of gL and either the cysteine 351 of gO (TC) or the cysteine 162 of pUL128 (PC), thus providing a molecular explanation for the modulation of cell tropism by these two glycoprotein complexes [62]. EM studies further confirmed the biochemical findings, showing that gO and pUL128L bind to the same site of the gL N terminus [62]. The UL148 gene product was found to modulate HCMV tropism by regulating the composition of alternative gH/gL complexes. In particular, the UL148 gene product is required to sustain high levels of gH/gL/gO, whereas UL148-null virus has defects in gH/gL/gO maturation and shows enhanced infectivity of epithelial cells [63].

Using the same purified soluble forms of TC and PC employed in defining their molecular architecture, the binding sites for the potently neutralizing human mAbs mentioned above [59] were defined on their surfaces [64]. By means of mass spectrometry paired with chemical crosslinking, three neutralizing epitopes were identified in gH (conserved in both TC and PC), along with five different neutralizing sites that were determined in the UL128L portion of PC [64]. In addition, EM and 3D reconstructions identified two distinct and opposite surfaces that were reactive with neutralizing mAbs in the pUL128L region of the PC.

TC and PC were repeatedly confirmed as mutually-exclusive viral entry complexes required for infection of fibroblasts and epithelial cells/endothelial cells, respectively. However, the gH/gL/gO complex was also found to be necessary for entry into epithelial cells/endothelial cells, as well as fibroblast cells [65,66,67]. This was documented by the observation that virions that were rich in PC, but poor in TC were poorly infectious also for epithelial cells/endothelial cells. This finding was interpreted as the result of the missing fusion machinery activation in the absence of gH/gL/gO, which promotes the gB-mediated fusion of the virus envelope and cell membrane in fibroblasts, as well as the viral envelope and endosomal cell membrane in epithelial cells/endothelial cells [68]. At that time, the trigger for the interaction between gH/gL/gO and gB was assumed to be the binding of the PC to an as yet unknown cellular receptor. However, another group reported that HCMV gB and gH/gL alone form stable gB-gH/gL complexes [69].

## 9. Identification of Cellular Receptor(s) for HCMV TC and PC 

By 2015, a series of discoveries had resulted in a thorough characterization of the PC and TC architecture. Finally, in the three-year period between 2016 and 2018, the cellular receptors for these complexes were identified as well, thus paving the way for the study of HCMV infection/disease pathogenesis and the adoption of adequate measures for prevention and the development of an effective HCMV vaccine.

### 9.1. Identification of the Cellular Receptor for HCMV TC in Fibroblasts 

Although it had been known for several years that the TC is required for HCMV infection of fibroblasts, the cellular receptor for the TC was not identified until 2016, when Perez and colleagues demonstrated that the platelet-derived growth factor-α receptor (PDGFRα) is a cellular receptor for HCMV gH/gL/gO TC [68]. Consistent with a previous study reporting that PDGFRα activation is required for HCMV infection [70], it was discovered that the virus phosphorylated PDGFRα in fibroblasts, but not in epithelial cells, which lack PDGFRα. This was achieved by investigating the activation of receptor tyrosine kinases (RTKs) following exposure of epithelial cells (ARPE-19) or fibroblasts to HCMV. Using the recombinant TC and PC, immunoprecipitation studies coupled with mass spectrometry revealed that the gH/gL/gO TC directly interacted with PDGFRα, thus establishing the identity of a cellular receptor for TC. 

To further investigate the role played by PDGFRα in HCMV entry, a genetic approach was adopted, as follows: (i) downregulation of PDGFRα by short hairpin RNA markedly inhibited infection of fibroblasts by the PC-mutated viral strain AD169, but did not affect fibroblast infection by the wild-type VR1814; (ii) accordingly, a CRISPR-Cas9-mediated knockout of PDGFRα markedly reduced infection by AD169, but not the VR1814 HCMV strain, in the haploid cell line HAP-1; (iii) finally, using a lentiviral-mediated overexpression approach, it was found that the overexpression of PDGFRα in epithelial cells made the cells susceptible to AD169 infection, simultaneously increasing infection by VR1814. Taken together, these results showed that wild-type VR1814 uses two alternative pathways for entry into cells; one is dependent on PDGFRα, and the other appears to involve the ErbB family members (exploited by the PC; see Section 9.2 below), whereas the mutated AD169 virus relies only on PDGFRα.

In this study, the role of PDGFRα in virus entry was confirmed by two independent observations. Firstly, both gH-specific antibodies and the recombinant soluble TC inhibited viral infection of fibroblasts by AD169. Similarly, the mechanism of viral entry into epithelial cells was inhibited by anti-PC antibodies, as well as by the soluble PC. In addition, it was found that the anti-gO antibody was able to reduce ARPE-19 cell infection, thus suggesting that the TC may exert a dual function of viral ligand and activator of gB fusogenic activity [61,69]. Secondly, a high-affinity molecular interaction between TC and PDGFRα was found by using surface plasmon resonance and biolayer interferometry, while gO, but not gH/gL, was found to be essential for binding to PDGFRα.

**Figure 2:**
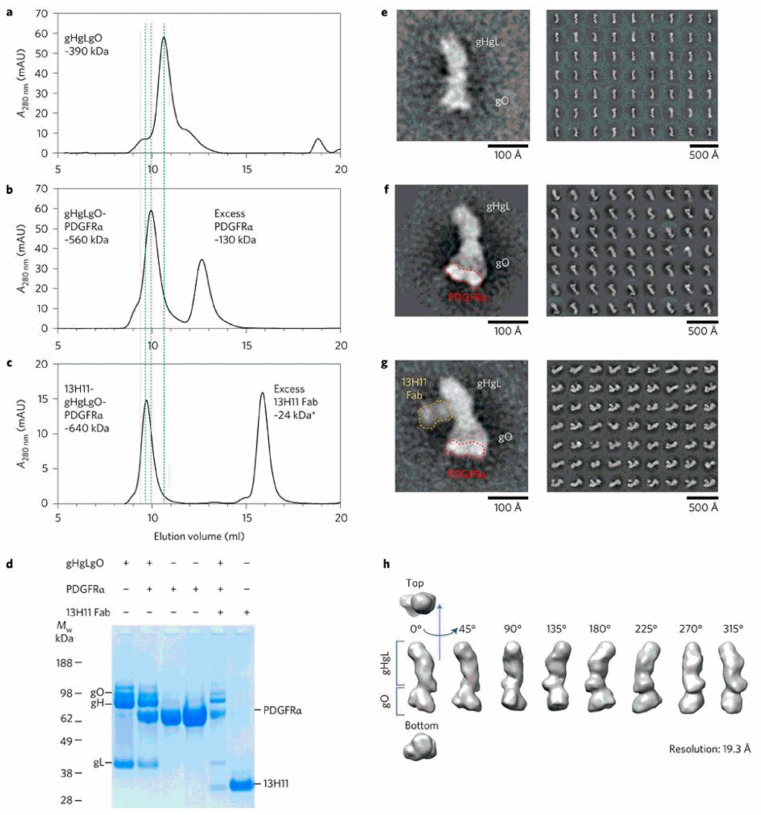
Analysis of the gH/gL/gO-PDGFRα complex. Size exclusion chromatography profile of the gH/gL/gO alone (**a**), or complexed with PDGFRα (**b**), or PDGFRα and the Fab fragment of the anti-gH mAb 13H11 (**c**). The shift in the elution time of (**b**) and (**c**) complexes compared to (**a**) is indicated by vertical dotted lines. (d) SDS-PAGE in reducing conditions of individual proteins and complexes, as indicated. (**e**–**g**) EM negative staining of gH/gL/gO alone (**e**), complexed with PDGFRα (indicated by a red dotted line) bound to gO (**f**), and with PDGFRα (indicated by a red dotted line) and the Fab of mAb anti-gH 13H11 (indicated by a yellow dotted line) bound to the twisted region of gH (**g**). (**h**) EM 3D map of gH/gL/gO at 19.3 Å resolution (from [68]).

The gH/gL/gO-PDGFRα interaction, subsequently confirmed in other studies [71,72], was further investigated by size-exclusion chromatography and EM. Size-exclusion chromatography revealed a high molecular weight complex formation when PDGFRα was added to the TC, which was blocked by a Fab fragment of the anti-gH human mAb 13H11 (Figure 2) [68]. SDS-PAGE confirmed the composition of different complexes, which were then analyzed by transmission EM and negative staining with two-dimensional averaging. This approach revealed that the TC has a boot-shaped conformation, where gH/gL has an elongated vertical structure with gO being placed in the horizontal angled extension [68]. From this imaging, it was possible to confirm and conclude that only gO interacts with PDGFRα. In addition, it was shown that the 13H11 Fab bound to a site of gH far away from gO, thus documenting that the neutralizing activity of mAb 13H11 did not target the TC–PDGFRα interaction, but was likely to interfere with the capacity of the complex to trigger the fusogenic activity of gB at the plasma membrane [73], as suggested by others [67,69]. It has also been reported that HCMV uses two different routes to enter ARPE-19 epithelial cells [74], i.e., virions produced in epithelial cells are thought to preferentially enter by fusion at the plasma membrane, whereas virions produced in fibroblasts enter by endocytosis.

### 9.2. Identification of the Cellular Receptor(s) for HCMV PC in Epithelial Cells/Endothelial Cells 

In 2018, by using a new, highly-sensitive and cell-independent receptor discovery platform, the cellular receptor for PC in epithelial cells/endothelial cells was finally identified as neuropilin2 (Nrp2), while PDGFRα was re-confirmed as an HCMV cellular receptor in fibroblasts [72]. A previous study observed the activation of the ErbB family member by the PC, but did not detect direct binding between the PC and ErbB receptors [68]. Thus, ErbB activation could be a consequence of the Nrp2 pathway activation upon direct engagement with the PC [72].

The identification of Nrp2 as an HCMV receptor was carried out by means of a new high-throughput methodology that allowed Ciferri and colleagues to study the interactions of human surface-expressed single transmembrane receptors with recombinant viral proteins, such as HCMV TC and PC, within a wide range of affinities (nM–µM) and with a high signal-to-noise ratio [72]. To this end, nearly all (n = 1297) single transmembrane receptors were cloned as extracellular domains and fused to a C-terminal human IgG1 Fc tag to allow binding of recombinant receptors to a ProteinA surface-coated or sensor-coated solid phase. To enhance the binding avidity, the C-terminal region of gH was replaced with the cartilage oligomeric matrix protein (COMP) scaffold to allow for TC/PC oligomerization, and an enzymatic reporter (β-lactamase) was used to detect viral protein binding to the immobilized single transmembrane panel. The subsequent methodological steps leading to the final identification of cellular receptors for both the TC and PC are as follows.

The system was validated by testing the TC, which showed high binding affinity to PDGFRα, as expected [68]. Then, the PC was assayed using the new test system, which revealed that Nrp2 was the main interaction partner for PC in the single transmembrane panel. Other single transmembrane receptors were found to have different degrees of affinity with either the TC (i.e., TGFβRIII and neuroregulin2 (NRG2)), or the PC (THBD, LILRB3, CD46, and FCAR). The role of these putative co-receptors needs to be further investigated. The results of the screening were confirmed by biolayer interferometry, which showed high-affinity binding of the TC to PDGFRα, as well as to TGFβRIII, but not to NRG2. Similarly, the PC was shown to bind to Nrp2, THBD, and LILRB3 (weak interaction), but not to FCAR. 

Following the isolation of Nrp2 from membranes of ARPE-19 epithelial cells, affinity purification (AP) and mass spectrometry (MS) were employed to confirm its binding to the PC complexed with the anti-gH-specific mAb 3G16 [59]. Furthermore, a flow cytometry analysis showed that an Nrp2 receptor (as well as TGFβRIII and CD46) was expressed on the cell surface of both epithelial cells/endothelial cells and fibroblasts. Thus, fibroblasts expressed similar levels of PDGFRα and Nrp2, which made fibroblasts more susceptible to HCMV infection compared to epithelial cells.

CRISPR/Cas 9-mediated gene mutagenesis was used to knock out Nrp2 in the near-haploid HAP-1 cell line and in human umbilical vein endothelial cells (HUVECs). In both cases, Nrp2 deficiency made the cells almost completely resistant to HCMV infection. 

Small interfering RNA (siRNA)-based knockdown of Nrp2 in ARPE-19 also markedly reduced HCMV infection. On the contrary, Nrp2 knockdown had only a limited effect on MRC-9 infection, which is consistent with the evidence that both Nrp2 and PDGFRα receptors can mediate fibroblast infection, with PDGFRα playing a major role.

**Figure 3:**
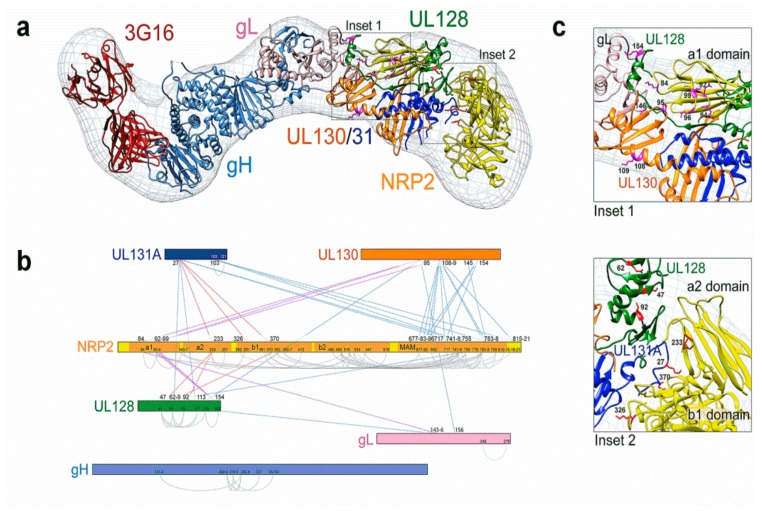
(**a**) Molecular architecture of the HCMV pentamer/Nrp2/mAb 3G16 Fab complex. (**b**) Several cross-links were observed between the Nrp2 a1 domain and residues of pUL130, pUL128, and gL. Additional cross-links were observed between the a2 and b1 domains and residues of pUL131 and pUL128. (**c**) Interactions between the HCMV pentamer complex (PC) and a2-b1-b2 domains of Nrp2 are observed in Insets 1 and 2 (from [72]).

The structure of the Pentamer-Nrp2 complex, bound to the Fab fragment of the neutralizing mAb 3G16 [59], was studied using negative staining EM and single particle analysis, which revealed the 3D model of interaction between the viral gp complex and its cellular receptor (Figure 3a) [72]. 

In addition, using chemical cross-linking coupled to MS analysis on the same samples that were used for EM analysis, the interaction of Nrp2 and PC was investigated (Figure 3b,c) [72]. This analysis detected several intra- and inter-molecular interactions within the Pentamer-Nrp2 complex. In particular, it showed that the four domains (a1-a2-b1-b2) of the extra-cellular region of Nrp2 interacted with distinct regions of the PC. Specifically, a1 mapped near the elbow formed by the V-shaped UL128L components of the PC, while the a2-b1-b2 domains occupied the larger densities emerging from the UL128L region of the PC. These data suggested that the a1 domain rearranges itself in relation to the remainder of Nrp2 upon PC binding. Moreover, the authors confirmed by SEC that the combination of all four extracellular domains of Nrp2 was required for PC binding, since neither Nrp2-a1a2 nor Nrp2-a1b1b2 alone were able to bind it. 

**Figure 4:**
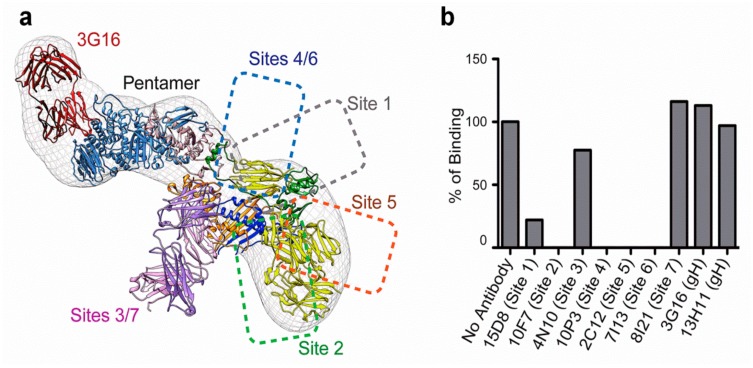
(**a**) Summary of the HCMV pentamer epitope mapping. (**b**) In vitro binding of neutralizing mAbs to pentamer epitopes, which, with the exception of mAbs targeting site 3/7, sterically block Nrp2 pentamer binding (from [72]).

Moreover, Ciferri and colleagues identified several amino acid residues on the PC UL128L subunits that were critical for Nrp2 (fused to an Fc) binding by using a set of 28 HCMV PCs, each carrying mutations in one of the UL128L subunits [72]. They showed that Nrp2 interacted with the PC at the binding sites of pUL128- (and pUL131)-specific human neutralizing mAbs (Figure 4a,b). Mutations in pUL128 or pUL131 dramatically reduced the interaction with Nrp2, whereas mutations of pUL130 had no effect on binding [72]. Overall, this experimental evidence provided an in-depth insight into the features of the architecture of the PC-Nrp2 complex.

### 9.3. Olfactory Receptor OR14I1 As An Additional PC Receptor of Epithelial Cells

The role of the olfactory receptor OR14I1 in HCMV infection of epithelial cells was recently established in a genome-wide CRISPR/Cas9 screen on epithelial cells expressing Cas9 and a single-guide RNA library [75]. The setup of the experimental study was based on the presumption that, by knocking out genes that are important for HCMV entry, cells will become resistant to infection and, hence, survive. To this end, epithelial cells were repeatedly exposed to HCMV for three months, and cellular clones resistant to viral infection underwent targeted sequencing, which identified OR14I1 (and to a lesser extent Nrp2) as the gene required for productive HCMV entry [75]. OR14I1 is a seven-domain transmembrane G-protein coupled receptor of the olfactory receptor family, which was found by Kowalik and colleagues to mediate HCMV attachment to epithelial cells’ surface and virus entry via endocytosis. In addition, they showed that OR14I1 downstream signaling mediated by the adenylate cyclase/protein kinase A/AKT pathway was required for HCMV infection [75]. However, no direct evidence of PC-OR14I1 binding (as observed for PC-Nrp2) has been reported yet, thus suggesting that OR14I1 is an additional HCMV receptor of epithelial cells.

## 10. Models for HCMV Entry into Fibroblasts and Epithelial Cells/Endothelial Cells 

### 10.1. Model for HCMV Entry into Fibroblasts

**Figure 5:**
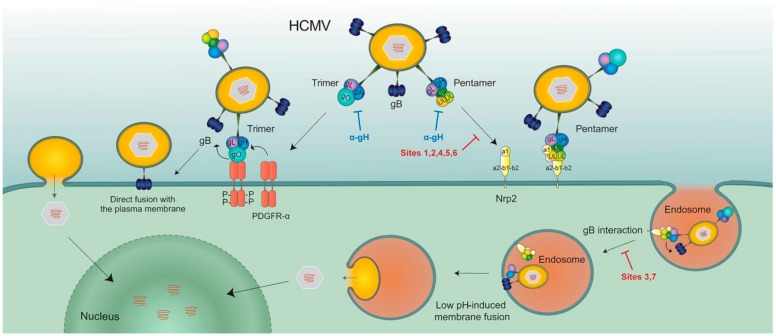
Models of HCMV entry into human cells using the trimer complex (TC) or PC complexes. The TC interacts with PDGFRα via gO, then gH/gL/gO activates the fusogenic activity of gB at the plasma membrane of human fibroblasts. Conversely, the PC first binds Nrp2, then the gH/gL component of the PC (or gH/gL/gO) induces endocytosis of virus particles [71], which are released into cytoplasm from endosomal membranes, following low-pH-induced activation of the gB fusion machinery (from [72]).

Following binding of the HCMV trimer gH/gL/gO to its cellular receptor PDGFRα, gH/gL/gO activates the fusogenic activity of gB, thus inducing fusion of the viral envelope with the cell membrane using a pH-independent pathway, finally resulting in productive HCMV infection of fibroblasts (Figure 5).

### 10.2. Model for HCMV Entry into Epithelial Cells/Endothelial Cells

PC binding to Nrp2, and possibly to OR14I1, appears to promote endocytosis of virus particles into epithelial cells/endothelial cells. Then, following gB activation by the gH/gL component of the PC (or gH/gL/gO), the release of virus particles from the endosomal compartment into the cell may be triggered by acidification of the vesicle (Figure 5) [72]. Alternatively, as recently reported [76], endocytosis of virions may be induced in an Nrp2-independent manner following gH/gL/gO interaction with an as yet unknown cellular surface receptor, followed by interaction of the PC with Nrp2. In other words, it is likely that both TC and PC are required for infection of epithelial cells/endothelial cells, but the emerging hypothesis is that the gH/gL component of both TC and PC serves as the first activator of the fusogenic activity of gB through a conformational change in HCMV gL occurring upon receptor binding [77], similar to that reported for Epstein-Barr virus gL [78,79]. We also find it intriguing that HCMV would use two pathways (one pH-dependent and the other pH-independent) to infect different cell types. In addition, PC-containing virus strains can infect PDGFRα-null fibroblasts because of the documented presence of an Nrp2 receptor on fibroblasts [72]. Thus, PC-Nrp2 interaction may be an alternative pathway for fibroblast infection.

## 11. Conclusions

The identification of HCMV glycoprotein complexes and the cellular receptors involved in virus entry is fundamental in understanding the mechanisms of HCMV infection and neutralization. This has prompted the development of novel strategies for the treatment and prevention of HCMV infection. In particular, the identification of the PC as a major determinant of HCMV cellular tropism and the most important target of human neutralizing antibodies supported the therapeutic use of the anti-PC antibody and the inclusion of the PC in vaccine formulations. Recently, a phase II clinical trial showed that the administration of a combination of two mAbs (one human to gH and one murine humanized to PC) reduced HCMV infection and disease in high-risk kidney transplant recipients [80].

Currently, the most advanced vaccine approach to exploit the new knowledge on PC function is a live attenuated AD169 whole virion vaccine (V160), in which PC expression was restored by repairing a frameshift mutation in the UL131 gene [81]. A phase II clinical trial of V160 in healthy seronegative women is ongoing (https://clinicaltrials.gov NCT03486834), while an mRNA based vaccine containing the PC and gB (mRNA-1647) is in a phase I trial (https://clinicaltrials.gov NCT03382405).

Other promising vaccine formulations are at the preclinical evaluation stage. One example is a subunit recombinant PC vaccine that was produced in a secreted form from the hamster ovary cell line, which was stably transfected with a single polycistronic vector encoding the five PC genes; in mice, this was found to induce neutralizing antibody titers that were 100–1000-fold higher than those induced by natural infection in humans [82]. More recently, a modified vaccinia virus Ankara-vectored vaccine composed of pp65, gB, and PC was evaluated in mice. The multi-antigenic vaccine elicited potent humoral immune responses that neutralized different HCMV strains, exhibiting also an antibody-dependent cellular cytotoxicity and polyfunctional T cell responses [83].
